# A Case of Purpuric Papulopustular Eruption on the Extremities Developed During Erlotinib and Ramucirumab Combination Treatment, Resulting in Complete Regression Without Oral Prednisone or Discontinuing Chemotherapy

**DOI:** 10.7759/cureus.76601

**Published:** 2024-12-30

**Authors:** Yoshihito Mima, Tsutomu Ohtsuka, Akihiro Tsujita, Yoshimasa Nakazato, Yuta Norimatsu

**Affiliations:** 1 Department of Dermatology, Tokyo Metropolitan Police Hospital, Tokyo, JPN; 2 Department of Dermatology, International University of Health and Welfare Hospital, Tochigi, JPN; 3 Department of Respiratory Medicine, International University of Health and Welfare Hospital, Tochigi, JPN; 4 Department of Diagnostic Pathology, International University of Health and Welfare Hospital, Tochigi, JPN; 5 Department of Dermatology, International University of Health and Welfare Narita Hospital, Narita, JPN

**Keywords:** chemotherapy discontinuation, combination treatment, complete regression, drug eruption, erlotinib, purpuric papulopustular eruption, ramucirumab

## Abstract

A 53-year-old woman undergoing combination therapy with epidermal growth factor receptor (EGFR) and vascular endothelial growth factor receptor (VEGFR) inhibitors for advanced lung cancer with brain metastases developed pustules and punctate purpura on both lower extremities. Histopathological examination revealed neutrophilic infiltration around the hair follicles and erythrocyte extravasation in the perivascular regions near the hair roots, leading to a diagnosis of purpuric papulopustular eruptions. The rash improved with oral doxycycline (100 mg/day) and topical corticosteroids. This case demonstrates that extensive purpuric drug eruptions without symptoms of vasculitis can be effectively managed with oral antibiotics, without the need for chemotherapy discontinuation or systemic corticosteroids. EGFR inhibitors can induce purpuric papulopustular eruptions through follicular occlusion and damage to vascular endothelial cells via inflammatory cells. In our case, the treatment duration was longer than previously reported, suggesting that VEGFR inhibitors delay wound healing and endothelial cell repair, potentially contributing to the development of purpuric papulopustular eruptions. As combination therapy with EGFR and VEGFR inhibitors was only introduced in 2022, to the best of our knowledge, the present case is the first of purpuric papulopustular eruptions occurring during this treatment regimen.

## Introduction

The combination therapy of epidermal growth factor receptor (EGFR) inhibitors and vascular endothelial growth factor receptor (VEGFR) inhibitors has been used to treat non-small cell lung cancer since 2022 [1]. EGFR inhibitors block signaling pathways involved in cancer cell proliferation and survival, as well as host-dependent processes that promote cancer growth [2]. EGFR is expressed in basal epidermal cells, sebocytes, and the outer root sheath epithelium, regulating the proliferation and differentiation of epidermal cells [3]. As a result, EGFR inhibitors can lead to various cutaneous symptoms by disrupting these functions [3]. Skin disorders, such as papulopustular eruption (acneiform rash), occur in over 90% of cases, whereas vasculitis, purpura, or erosive pustular dermatosis are rarely reported [4,5]. Purpura induced by EGFR inhibitors appears regardless of follicle regions, whereas papulopustular eruption typically arises from hair follicles [6,7]. VEGFR inhibitors, which are fully human immunoglobulin G1 monoclonal antibodies, bind with high affinity to the VEGFR2 extracellular domain, regulating endothelial cell proliferation [8]. Although VEGFR inhibitors increase the risk of internal bleeding and thrombosis, no skin symptoms, such as purpura or vasculitis, have been reported [8]. Cases of chemotherapy-associated purpuric papulopustular eruption have rarely been reported [9]. Herein, we report a case of follicular purpuric papulopustular eruption on the lower extremities that developed during treatment with erlotinib (an EGFR inhibitor) and ramucirumab (a VEGFR inhibitor), which improved with topical betamethasone and oral doxycycline.

## Case presentation

**Figure 1:**
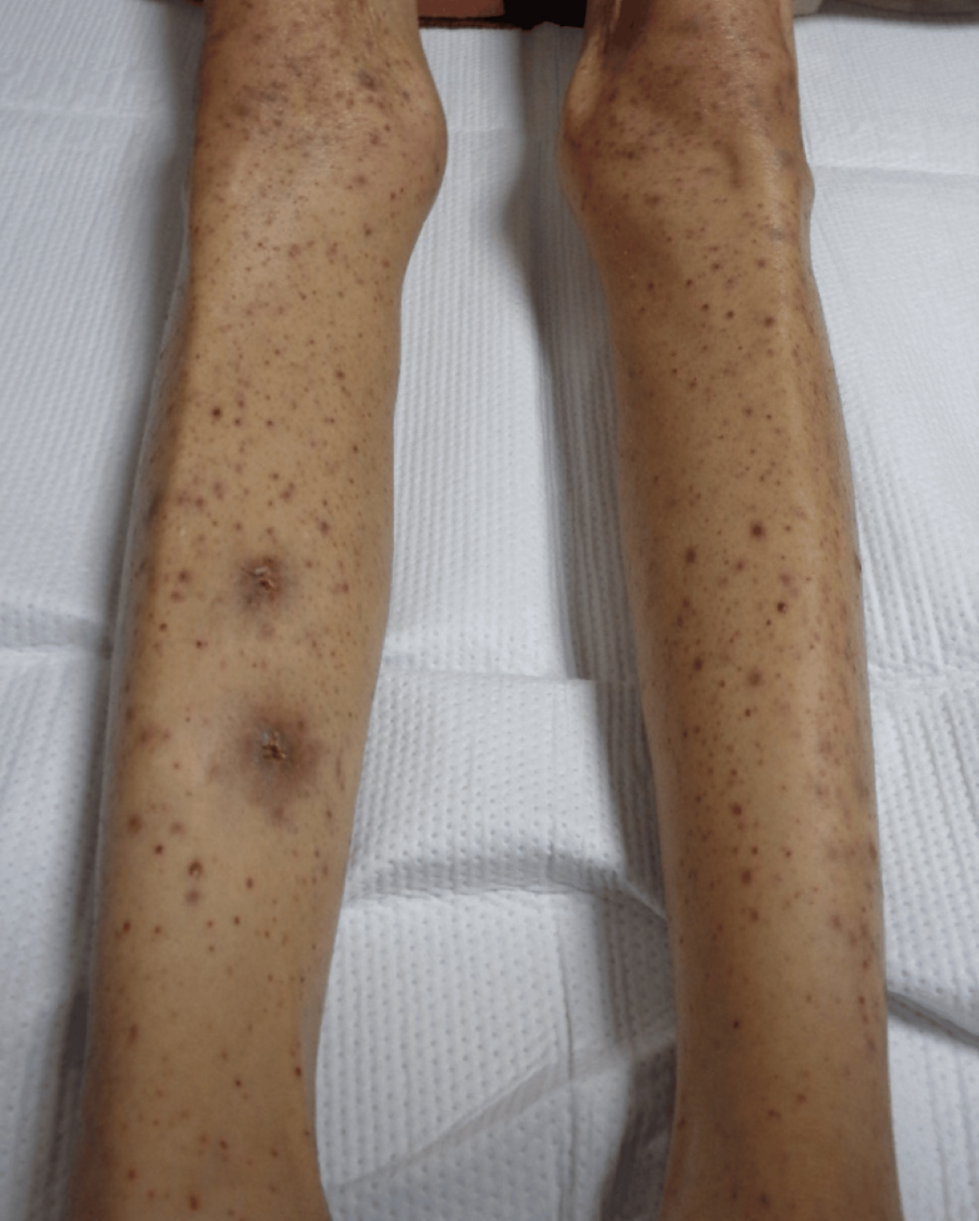
Multiple pustules and punctate papular purpura observed in both lower limbs.

A 53-year-old woman received a diagnosis of advanced lung cancer and brain metastases. She initially received osimertinib (an EGFR inhibitor) as adjuvant chemotherapy owing to her EGFR L858R mutation. After 5 months, the lung cancer progressed, prompting a switch to a combination of erlotinib (another EGFR inhibitor) and ramucirumab (a VEGFR inhibitor). After 1 month on this regimen, she developed multiple pustules and punctate purpura, which were refractory to gentamicin ointment, leading to her referral to our department. The patient had been taking lansoprazole, telmisartan, celecoxib, and rebamipide for over 4 years, with no new medications introduced following her cancer diagnosis. Physical examination revealed pustules and punctate papular purpura on both lower limbs, suggesting leukocytoclastic vasculitis (Figure 1).

**Figure 2:**
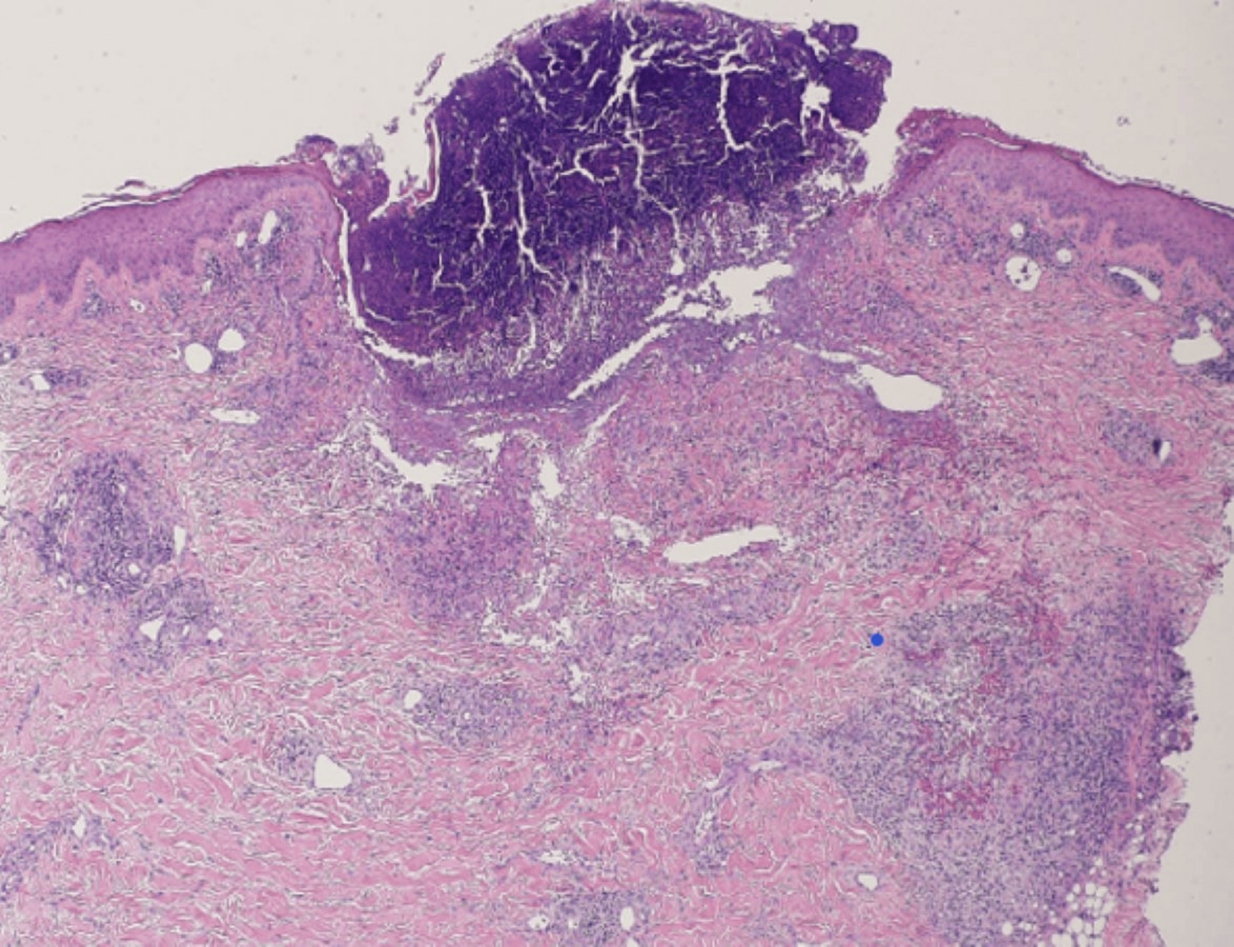
Histological examination of a skin biopsy specimen showing crusts composed of necrotic material at the center (hematoxylin and eosin stain (HE) ×40).

**Figure 3:**
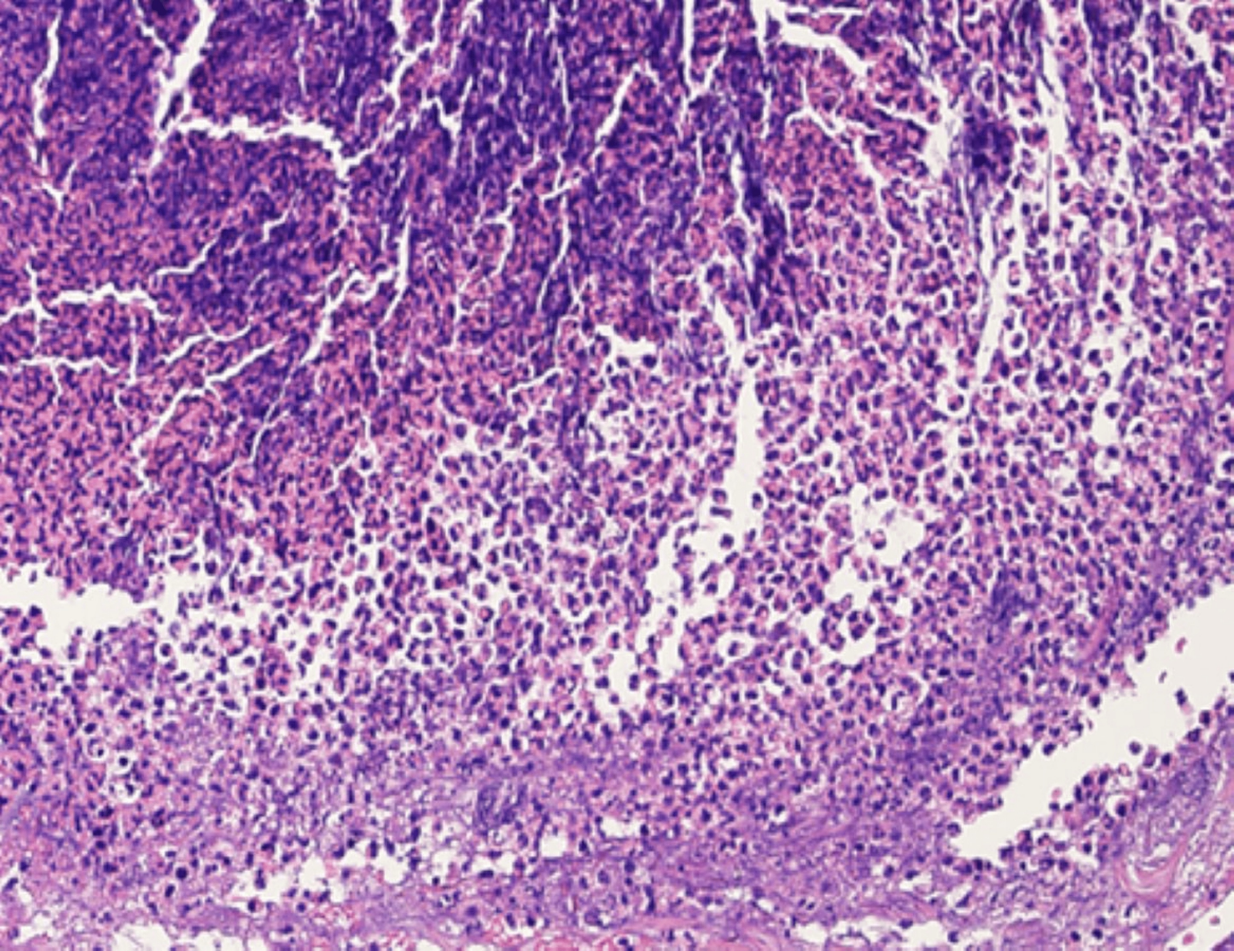
Histological examination of a skin biopsy specimen showing crusts composed of necrotic material at the center, inflammatory cell infiltration (HE ×100).

**Figure 4:**
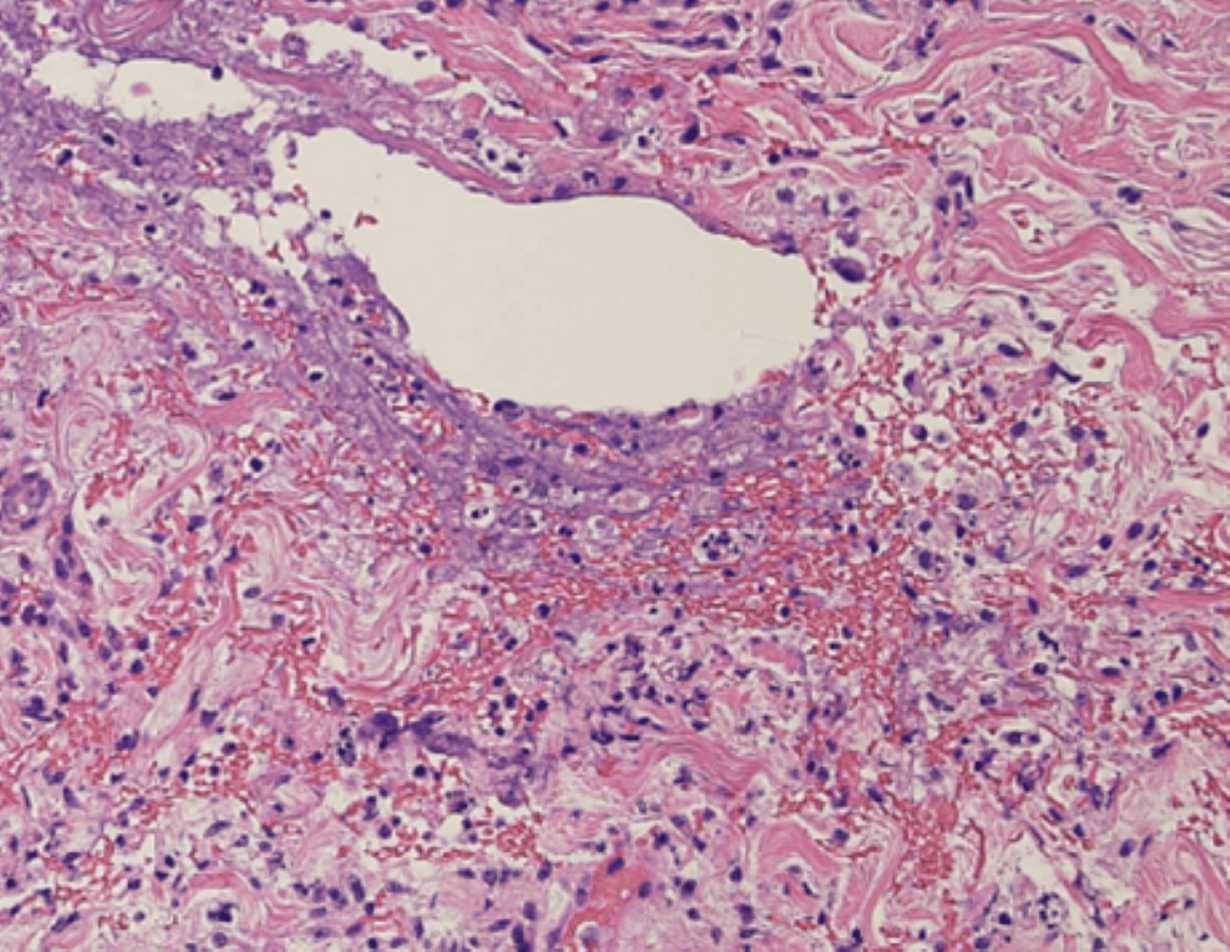
Histological examination of a skin biopsy specimen showing extravascular erythrocyte leakage near hair follicles in the superficial dermis (HE ×100).

Repeated bacterial cultures from the pustules were negative, suggesting chemotherapy-induced papulopustular eruption (acneiform rash). Blood tests showed a normal platelet count (334 × 10³/μL) and a slight increase in D-dimer (2.00 μg/mL (normal range: 0-1.00)). All markers for collagen diseases or vasculitis were negative. Histopathological examination of the punctate papular purpura revealed a crust with hair follicle structures in the dermis below it, notable neutrophil infiltration around the hair follicles, and erythrocyte leakage near the vessels, with no perivascular neutrophil infiltration, vessel wall thickening, or fibrinoid degeneration (Figures 2-4).

**Figure 5:**
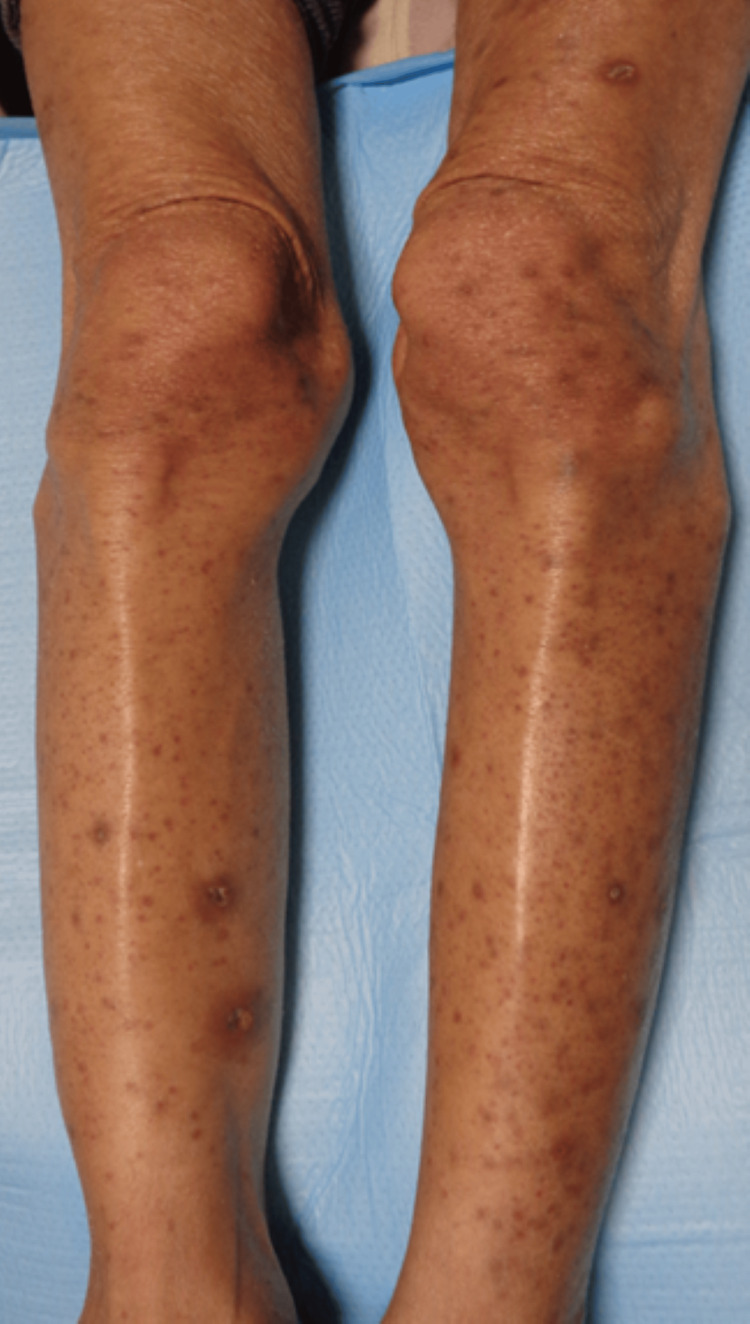
Pigmented punctate purpura and refractory papulopustular eruption on the lower limbs.

**Figure 6:**
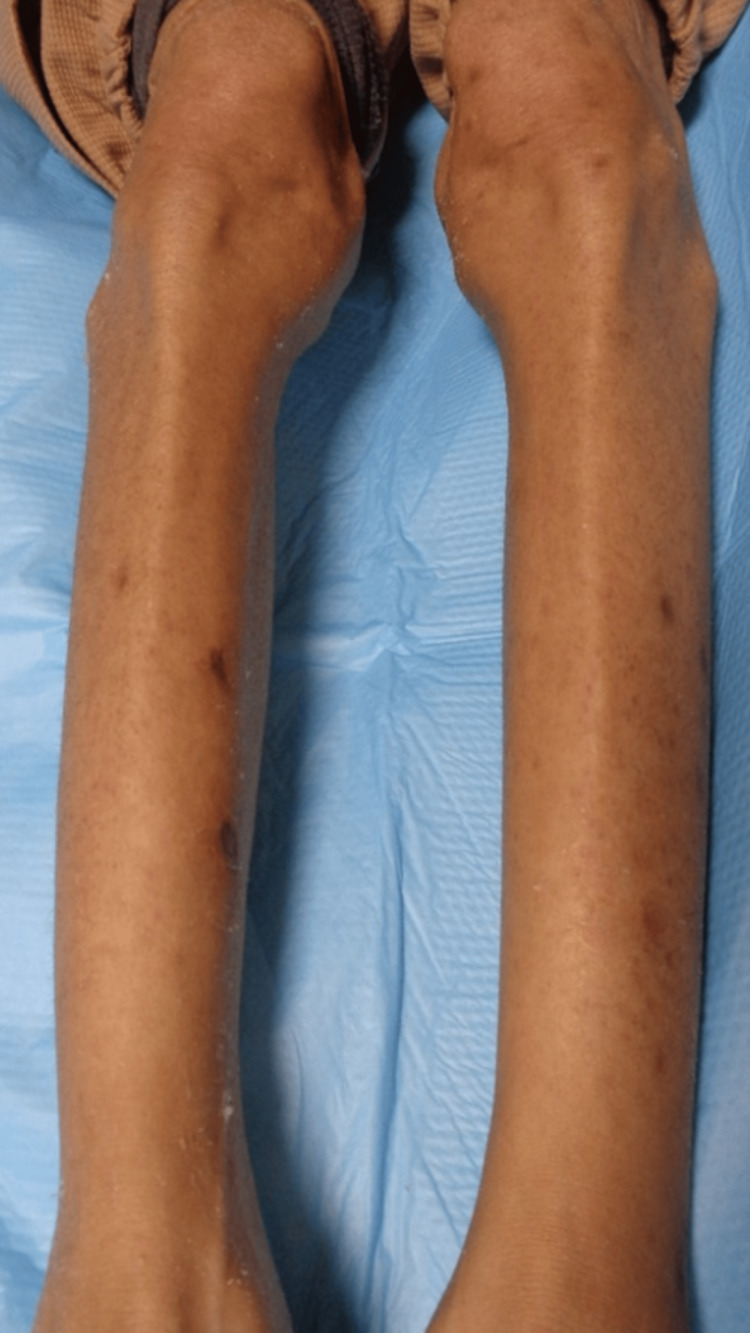
The pigmented punctate purpura and papulopustular eruption show complete regression.

Immunofluorescence staining results were negative. Based on a previous report [9], she was diagnosed with follicular purpuric papulopustular eruption, in conjunction with papulopustular eruption and punctate purpura. We considered the possibility that erlotinib and ramucirumab induced the rash, given her clinical course and a prior report [9]. Owing to the extensive coverage of the rash, we considered discontinuing chemotherapy and initiating oral prednisone. However, respecting the patient’s desire to continue the same chemotherapy regimen and considering the absence of systemic symptoms or leukocytoclastic vasculitis, we opted for oral doxycycline (100 mg/day) and topical betamethasone dipropionate, classified as a very strong corticosteroid, for anti-inflammatory treatment without initiating oral prednisone or discontinuing chemotherapy. After 2 months, the purpura regressed with pigmentation, but the papulopustular eruption remained refractory (Figure 5). By 4 months, the follicular purpuric papulopustular eruption had completely resolved (Figure 6).

## Discussion

Papulopustular eruptions (acneiform eruptions) are thought to arise from follicular occlusion due to comedones, resulting from inhibited EGFR signaling. This promotes inflammatory cell infiltration, increases inflammatory cytokine production, and leads to epidermal necrosis [10]. Doxycycline not only inhibits bacterial protein synthesis to suppress bacterial infections in acneiform lesions but also broadly suppresses the activity of inflammatory cytokines, matrix metalloproteinases, and immune cells such as neutrophils [11]. Through its combined antibacterial and anti-inflammatory properties, doxycycline is considered effective for treating acneiform eruptions [11]. Indeed, a systematic review of acneiform eruptions associated with EGFR inhibitors suggests that doxycycline is the most effective prophylactic antibiotic [12]. Doxycycline, alongside topical corticosteroids, is considered an effective treatment option for these eruptions [13].

For EGFR inhibitor-induced purpura, either the inhibitor or inflammatory cells activated by it may directly damage vascular endothelial cells, leading to purpura [14]. Additionally, purpura development may involve increased vascular tone and permeability due to inhibited EGFR in perivascular smooth muscle [15]. Anti-staphylococcal antibiotics are effective in treating drug-induced purpuric eruptions [6,16]. Research on skin microbiomes indicates a link between purpuric eruptions from EGFR inhibitors and the abundance of Staphylococcus, suggesting its role in the eruptions' pathogenesis [6,16]. Moreover, a decrease in epidermal β-defensin 2 and filaggrin in these eruptions implicates dryness and compromised skin barrier function [16]. Prior case reports have identified xerotic eczema beneath purpuric eruptions [9], underscoring the need for moisturizers to support skin barrier function during EGFR inhibitor therapy [6,9,16]. In this case, the patient had a history of dry skin, so moisturizers were applied after the purpuric papulopustular eruptions improved.

Typically, purpura induced by EGFR inhibitors can occur regardless of the follicular region [6,7]. However, in our case, purpura developed at the same site as the follicular pustules, resulting in a purpuric papulopustular eruption. Although only one other case of purpuric papulopustular eruption linked to EGFR inhibitors has been reported [9], our case may represent the first occurrence during combination therapy with EGFR and VEGFR inhibitors.

Previous reports indicate that EGFR inhibitor-induced papulopustular eruptions typically improve within a few weeks to months with treatment [1-4]. In our case, however, improvement took 5 months, suggesting a refractory nature due to wound-healing impairment caused by ramucirumab [8]. Although purpura associated with EGFR inhibitors tends to resolve within a few weeks of initiating antimicrobial therapy [6,9], our case required 2 months of doxycycline treatment. The VEGFR2 inhibitor may contribute to prolonged treatment duration by delaying the repair of vascular endothelial cells and causing defects in the plasma membrane or underlying matrix, contributing to the development of purpuric papulopustular eruptions alongside EGFR inhibitors [8]. Given the extended treatment for both papulopustular and purpuric eruptions, VEGFR inhibitors may also play a role in the development of purpuric papulopustular eruptions. In addition to oral doxycycline, the use of topical steroids may have contributed to the improvement of purpuric papulopustular eruptions by alleviating inflammation around the follicles and suppressing the functions of anti-EGFR and anti-VEGFR2.

In cases of EGFR inhibitor-induced vasculitis, discontinuing or reducing chemotherapy or introducing oral steroids should be considered [17,18]. Conversely, many cases of EGFR inhibitor-associated purpura, including ours, have improved with antimicrobial therapy without the halt of chemotherapy or the initiation of oral steroids [6,9]. Discontinuing chemotherapy may negatively impact patient prognosis, making it advantageous to manage non-vasculitic purpuric drug eruptions while continuing treatment.

We reported a rare case of follicular purpuric papulopustular eruption associated with erlotinib and ramucirumab. As this chemotherapy combination started in 2022, reports of similar eruptions are limited [1,9]. Further research and case accumulation are needed to clarify the mechanisms behind skin adverse effects associated with erlotinib and ramucirumab.

## Conclusions

We report a case of purpuric papulopustular eruptions occurring during combination therapy with EGFR and VEGFR inhibitors. While differentiation from vasculitis with systemic symptoms is essential, purpuric papulopustular eruptions may be effectively managed with oral antibiotics without the need for chemotherapy discontinuation or systemic corticosteroids. Combination therapy with EGFR and VEGFR inhibitors was only introduced in 2022, and further accumulation of cases and studies is needed to enhance our understanding of purpuric papulopustular eruptions associated with this treatment regimen.
